# Linking Evidence-Based Program Participant Data with Medicare Data: The Consenting Process and Correlates of Retrospective Participant Consents

**DOI:** 10.3389/fpubh.2014.00176

**Published:** 2015-04-27

**Authors:** Philip Lloyd Ritter, Marcia G. Ory, Matthew Lee Smith, Luohua Jiang, Audrey Alonis, Diana D. Laurent, Kate Lorig

**Affiliations:** ^1^Stanford Patient Education Research Center, Stanford School of Medicine, Stanford, CA, USA; ^2^School of Public Health, Texas A&M University, College Station, TX, USA; ^3^College of Public Health, The University of Georgia, Athens, GA, USA; ^4^Department of Epidemiology & Biostatistics, School of Public Health, Texas A&M Health Science Center, College Station, TX, USA

**Keywords:** chronic disease self-management, patient education, Medicare, consenting, cost analysis

## Abstract

As part of a nation-wide study of the Chronic Disease Self-Management Program (*National Study*), older participants were asked to consent to have their Medicare data matched with study data. This provided an opportunity to examine the consenting process and compare consenters, refusers, and non-responders. We compared the three groups on a large number of variables. These included demographic, *National Study* participation, health indicator, health behavior, and health-care utilization variables. We assessed differences in 6-month change scores for time-varying variables. We also examined whether asking participants to consent prior to the final questionnaire impacted completion of that questionnaire. Of 616 possible participants, 42% consented, 44% refused, and 14% failed to respond. Differences by ethnicity were found, with Hispanics more likely to consent. There was a consistent tendency for those who participated most in the *National Study* to consent. With the exception of number of chronic diseases, there was no evidence of health indicators or health behaviors being associated with consenting. Participants with more physician visits and more nights in the hospital were also more likely to consent. Those asked to consent before the 12-month follow-up questionnaire were less likely to complete that questionnaire than those who were asked after. Fewer than half consented to link to their Medicare data. The greater willingness to consent by those who participated most suggests that willingness to consent may be part of program engagement. Consenters had more diseases, more MD visits, and more nights in the hospital, suggesting that greater contact with the medical system may be associated with willingness to consent. This indicates that examinations of Medicare data based only on those willing to consent could introduce bias. Asking for consent appears to reduce participation in the larger study.

## Introduction

Most of what we know about the effectiveness of evidence-based chronic disease self-management programs (CDSMP) comes from self-reports of health and health-care outcomes experienced by participants (1). In 2010, the National Council on Aging (NCOA), the Stanford Patient Education Research Center and Texas A&M Health Science Center’s Program on Healthy Aging initiated a major longitudinal nation-wide U.S. study of participants in a dissemination of the Stanford CDSMP (2). The primary purposes of the project were to inform NCOA’s technical assistance work and assessing the impact of the program when offered in a variety of “real world” settings across the nation. Baseline enrollment of study participants began in August 2010 and ended in April 2011, with subsequent collection of 6- and 12-month follow-up survey data. That study is known as the *U.S. National Study of the CDSMP* (referred to as the *National Study*), and details of the intervention and the self-reported outcomes have been published elsewhere (3, 4, 5, Ory et al. in prepartion).

After completion of the initial intervention and during the collection of follow-up questionnaires, the Centers for Medicare & Medicaid Services (CMS) contracted with NCOA for a pilot study to examine the feasibility of matching *National Study* participants with their CMS data. Linking with administrative claims data would provide an alternative and potentially more precise method for examination of health-care utilization and associated costs savings attributed to program participation.

Because consent to match study data with CMS data was not obtained at the beginning of the *National Study*, all potential subjects had to provide supplemental consent for the specific purposes of having their CDSMP data linked to CMS Medicare Administrative Data. A subset of *National Study* participants who were at least 65.5 years of age at the beginning of the *National Study* were invited to enroll in the CMS study. This paper reports about this consenting process and how consenters differed from: (a) those who actively declined to participate (refusers); and (b) those who did not respond (non-responders). Institutional Review Board (IRB) approval was obtained at Stanford University and Texas A&M University for the initial National Study and for the subsequent consenting study reported in this paper.

## Materials and Methods

### Consenting protocol

There were several steps to the consenting process. We started by mailing consent requests to 188 participants who had recently completed their 6-month questionnaires and had been 65.5 or older at the beginning of the *National Study*. The process would continue as other participants completed or would have completed 6- or 12-month follow-up questionnaires. These first mailings occurred in August and September 2011. Potential participants were asked to provide the last four digits of their social security number (SSN) and to consent to allow their study identifying information to be used to obtain Medicare claims data. There was an initial assumption that having a partial SSN would accelerate the matching process. After 3 weeks and several follow-up or attempted follow-up contacts by telephone, only 23% of the initial 188 potential participants had consented. Feedback from participants revealed some concerns about providing SSNs. We therefore suspended the consenting process and modified the protocol for those who had not yet responded and for subsequent mailings. In the revised protocol, we asked participants for permission to match their study identifying data with their Medicare data using name, gender, address, and date of birth – four identifiers that we hypothesized would yield fairly accurate matches with CMS records. Detailed information on processes for linking various administrative data sets can be found elsewhere (6, 7). Given the low initial response rate and stated concerns among older adults about revealing such highly identifiable information, requests for any part of participants’ SSNs were dropped. The following six-step protocol was followed for the remainder of the study.

Step 1: Each potential participant received a short hand-addressed note explaining the CMS study and telling them that in a few days they would receive a gray envelope containing the study details, consent forms, and a small gift. The gray envelope was used so that the mailing could not be confused with the *National Study* questionnaires, which were sent out in white envelopes.
Step 2: Three days later consent forms were mailed along with a gift of four “forever” stamps.
Step 3: Five to 10 days later at least two calls were made. Messages were left on the second calls if participants were not yet reached.
Step 4: Two weeks later those who had not responded received a post card reminder.
Step 5: One month after the first mailing, those who had not responded received a second consent-form mailing.
Step 6: Approximately 6 weeks after the initial mailing, phone calls were made to participants. At least three attempts were made to reach each participant. Consents could be obtained on the phone if study participants allowed the research assistant to read the entire five-page consent statement prior to accepting via verbal consent.

### Data analyses

Primary analyses compare those who consented to participate in the CMS study with those who were eligible to participate (were enrolled or likely to be enrolled in Medicare) but did not consent. The latter group consisted of two subsets, those who actively declined to participant and those who did not respond to consent requests. Consequently, two additional sets of comparisons were conducted comparing: (1) those who consented to those who actively declined; and (2) those who either consented and/or declined (responded) to those who did not respond to the mailings and phone calls. Given the study emphasis on who would actively consent to have their data linked, only those able to give consents (e.g., living participants) were included in these analyses.

Comparisons between groups of individuals (consenters, refusers, and non-responders) were made using demographic, CDSMP workshop participation, health indicator, health behavior, and health-care utilization variables (described below). Differences between groups were tested using independent sample *t*-test for continuous variables, chi-squares for categorical variables, and non-parametric (Wilcoxon) tests for low frequency medical utilization variables.

The consent forms were first mailed to all potential participants after they had the opportunity to complete 6-month or, in the case of the earliest *National Study* recruitment cohorts, 12-month follow-up questionnaires. Thus, we were also able to examine whether 6-month changes were related to whether participants consented, refused, or did not respond. We compared mean changes on two health indicators, three health behaviors, and three health-care utilization measures.

### Measures

Demographic variables included age, gender, number of years of education, and ethnic identification (African-American, Hispanic, or non-Hispanic white). CDSMP program participation was measured in a number of ways. Both the mean number of workshop sessions attended (out of a possible six) and completion of the program (defined as having attended at least four of the six sessions) were tabulated. Assuming that those who had previously consented to be in a sub-study might differ from those who had not, we calculated the percentage of National Study participants who were also participating in a sub-study for people with Type 2 diabetes and had agreed to furnish blood samples for testing hemoglobin A1c levels (8). Finally, we tabulated the proportions of participants who completed 6- and 12-month follow-up questionnaires as part of the larger *National Study*.

Three health indicators were measured. These consisted of the mean number of comorbid conditions reported, PHQ-8 depression, and self-reported general health. The PHQ-8 consists of eight items, which are summed resulting in a range of 0–24 (9). The self-reported general health measure consists of a single-item ranging from one (excellent) to five (poor) and was originally used in the National Health Interview Survey (10). For each of the three measures, a higher score is less desirable (more conditions, greater depressive symptoms, and worse overall health).

The three health behaviors were whether exercised during the past week, communication with physician and medication adherence. The exercise measure was a single-item that asked if the participant had participated in physical activity or exercise within the last week. Communication with physician scale is a 3-item, 6-point scale and was developed to evaluate the CDSMP and related programs and has been described by Lorig and colleagues (11). Medication adherence was the sum of four questions regarding medication use (12). A higher score indicates less medication adherence.

We also examined three measures of health-care utilization: physician visits, emergency department visits, and nights of hospitalization in the previous 6-months. These self-report measures have been found to be relatively unbiased when compared to health provider records in an earlier study (13).

We calculated completion rates of 12-month follow-up questionnaires for those who were asked to consent before 12-month follow-up and those with consent forms sent after the 12-month follow-up period. This was to help ascertain if the consent process might have affected participation in follow-up within the larger study.

## Results

### Participants

**Figure 1:**
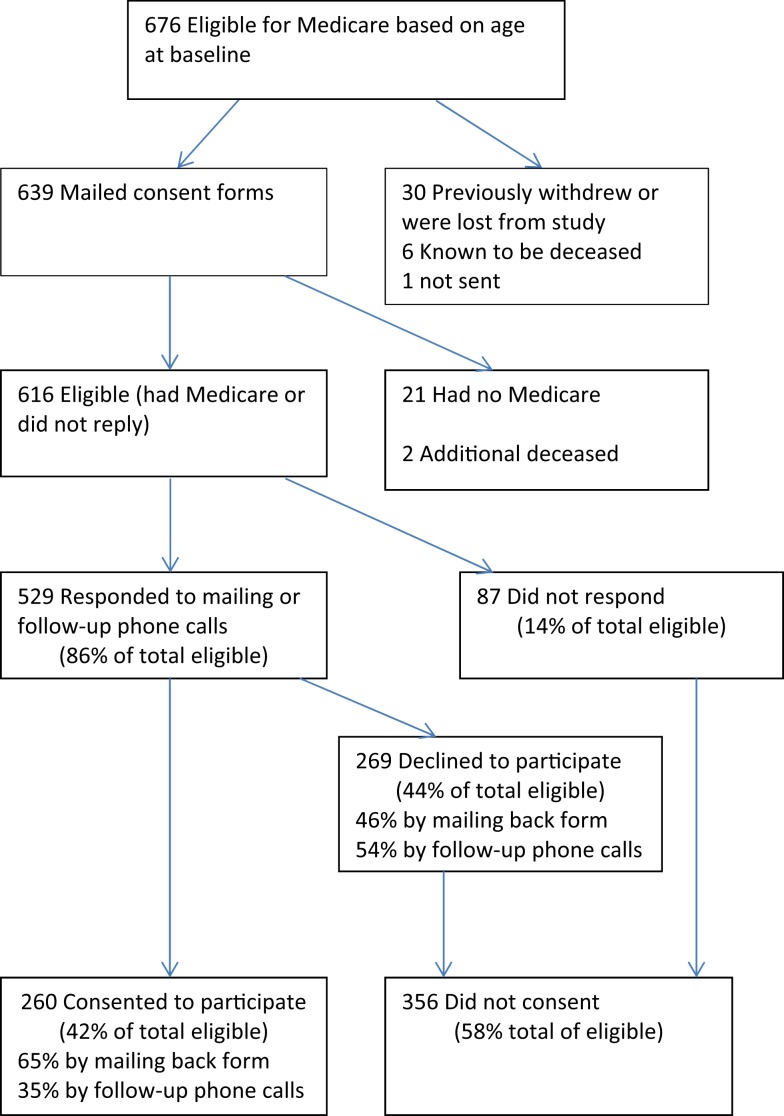
**Status of CDSMP participants invited to participate in Medicare cost study**.

At the time CMS consent requests and forms were mailed, there were a total of 639 *National Study* participants who were the appropriate age to have Medicare (65.5 or older). These people were mailed CMS consent requests between August and December 2011 (Figure 1). Of the 639, 21 subsequently indicated that they were not participating in Medicare for a variety of reasons but mainly because they were still employed and/or had other medical insurance, including veterans’ benefit. This left 618 participants with Medicare. An additional two had died before receiving the mailing, as had 6 participants who were known to have died before the mailing. The eight deceased individuals (six who were never sent consent forms and two who were) are not included in these analyses. Thus, there were 616 participants (618 minus the 2 who were discovered to have been deceased) who could have actively consented to participate. Of these, 260 consented, 169 by mail, and 91 by phone. Two-hundred sixty-nine actively declined, while 87 did not respond. In summary, of the 616 eligible participants, 42% consented to participate, 44% declined to participate, and 14% did not respond.

### Non-responders

Of the 87 who did not respond, 12 indicated they did not want to be called or hung up and were put on a “do-not-call” list. There were 20 participants who were contacted and indicated they would return the forms but never did. These included seven who mentioned being ill and seven who indicated they were very busy, including two with deaths in the family. Two thought they had returned the forms, four said they had not received the forms and four requested we call back but were not reached. The remaining 45 were never contacted (failed to respond to mail or phone messages, had no or full answering machines, or had no or disconnected phone numbers).

### Differences between consenters, refusers, and non-responders at baseline

Table 1 shows the mean of continuous measures or the percentage of categories at baseline for each of the three groups of potential CMS study participants. The last three columns present the P-values for three sets of comparisons. The first column compares those who consented with all those who did not consent (both refusers and non-responders). The second column shows results from the comparison of those who consented with those who actively declined to consent. The last column examines the comparison of those who responded with those who did not respond.

**Table 1:** **Baseline participant and workshop characteristics among CDSMP national study participants by consented, refused, or failed to respond to invitation to participate in Medicare cost study**.

Baseline characteristic	Consented (*N* = 260)	Refused (*N* = 269)	No response (*N* = 87)	*P*-value (A) consent versus no consent	*P*-value (B) consent versus decline	*P*-value (C) response versus no response
**DEMOGRAPHIC CHARACTERISTICS**						
Mean age	73.7 (5.07)	74.2 (5.08)	73.7 (5.17)	0.419	0.315	0.707
Mean years of education	13.1 (3.97)	13.2 (3.23)	12.6 (4.4)	0.997	0.623	0.261
Percent male	16.5%	16.7%	16.1%	0.991	0.953	0.900
Percent African-American	15.0%	12.6%	23.0%	0.388	0.432	0.057
Percent Hispanic	18.9%	9.29%	21.8%	*0.031*	*0.002*	0.098
Percent non-Hispanic White	61.5%	68.3%	47.6%	0.660	0.104	0.002
**WORKSHOP PARTICIPATION**						
Mean number of sessions attended (0–6)	4.92 (1.61)	4.23 (1.96)	4.13 (1.89)	<*0.001*	<*0.001*	*0.037*
Completed program (4 +)	85.5%	72.9%	69.0%	<*0.001*	<*0.001*	*0.033*
Participated in HbA1c study	12.7%	9.29%	5.75%	0.084	0.211	0.137
Completed 6-month questionnaire	95.0%	90.0%	48.3%	<*0.001*	*0.029*	<*0.001*
Completed 12-month questionnaire	92.3%	80.3%	35.6%	<*0.001*	<*0.001*	<*0.001*
**HEALTH INDICATORS**						
Number of Chronic diseases	3.03 (1.55)	2.78 (1.45)	2.44 (1.18)	*0.005*	0.052	*0.001*
PHQ depression	5.35 (4.56)	4.79 (4.43)	6.06 (5.10)	0.513	0.155	0.061
General health	3.07 (0.882)	2.99 (0.916)	3.14 (0.904)	0.516	0.282	0.302
**HEALTH BEHAVIORS**						
% Exercised (past week)	76.5%	76.1%	71.3%	0.809	0.908	0.313
Communication with MD	2.66 (1.33)	2.81 (1.32)	2.57 (1.46)	0.421	0.202	0.276
Medication adherence	0.808 (1.05)	0.732 (0.971)	0.779 (1.04)	0.440	0.392	0.935
**HEALTH-CARE UTILIZATION**						
# of physician visits	3.80 (3.54)	3.29 (3.20)	2.52 (3.07)	0.012	0.081	*0.009*
# ED visits	0.142 (0.411)	0.205 (0.610)	0.118 (0.359)	0.658	0.486	0.526
# of hospital nights	0.946 (4.08)	0.300 (1.02)	0.977 (4.90)	*0.045*	0.063	0.468

*For means, standard deviations are given in parentheses. Percentages are the percent within each of the three categories (consenters, refusers, and non-responders) that belong to the variable (e.g., 16.5% of consenters were male compared to 16.7% of refusers)*.

**P*-values are from chi-square test for categorical variables and from independent sample *t*-tests for continuous variables, except number of ED visits and number of hospital nights, which are from Wilcoxon rank sum tests. *P*(A) compares those who consented (*N* = 260) with all who did not consent (*N* = 356). *P*(B) compares those who consented (*N* = 260) with those who actively declined to consent (*N* = 269). *P*(C) compares those who responded (*N* = 529) with those who did not respond (*N* = 87)*.

*P-values less than 0.05 are shown in italics*.

Among the demographic variables, there was little difference in age, education, or gender. The non-response group had higher proportions of African-Americans and Hispanics. In addition, among those who responded, the consenters had greater proportions of African-Americans and Hispanics than did the refusers, although the differences were only marginally significant for African-Americans (*p* = 0.057). The proportion Hispanic was significantly higher for consenters when compared to both refusers and to all others. Described in another way (not shown in the table), Hispanics were more likely to consent than non-Hispanics (53 versus 40%, *p* = 0.026). African-Americans were more likely to not respond than non-African-Americans (22 versus 13%, *p* = 0.027). Non-Hispanic whites had the lowest level of non-response (11 versus 20% for others, *p* = 0.002).

There were a number of significant differences in workshop participation indicators. Consenters attended more sessions, were more likely to have completed the program and more likely to return 6- and 12-month questionnaires. Those who had already consented to participate in the diabetes A1c study were also more highly represented among consenters than non-consenters, but the difference was not statistically significant (*p* = 0.084).

The mean number of comorbid chronic conditions was greater among those who consented and lower among those who did not respond. The other two health indicators (depression and self-reported overall health) did not differ significantly among the three groups. Similarly, there were no statistical differences among baseline health behaviors.

There were two significant differences in baseline self-reported health-care utilization. Consenters had a higher mean number of physician visits in the last 6 months compared to all non-consenters and to non-responders. Consenters also had a higher number of hospital nights than those who did not consent.

### Six-month changes in health indicators, behaviors, and utilization

No significant differences were found in 6-month changes in the two health indicators (depression and self-reported overall health) and three health behaviors (exercise, communication with physician, and medication adherence) among the three groups (Table 2). Among health-care utilization measures, those who consented had a 6-month increase in emergency department visits compared to those who refused to consent or did not consent overall. Although not significant, non-responders had greater reductions in hospitalizations than did consenters, while those who actively refused slightly increased their nights of hospitalization. As noted above, non-response for consents was associated with lower return of 6-month questionnaires – only 48% of non-responders had completed 6-month questionnaires compared to 92% of responders (*p* < 0.001). Thus, the reduction in hospitalizations among the non-responders may reflect a biased subset of all non-responders.

**Table 2:** **Six-month changes, among CDSMP national study participants by consented, refused, or failed to respond to invitation to participate in Medicare cost study**.

Baseline measure	Consented (*N* = 246)	Refused (*N* = 241)	No response (*N* = 42)	*P*-value (A) consent versus no consent	*P*-value (B) consent versus decline	*P*-value (C) response versus no response
**HEALTH INDICATORS**						
PHQ depression	−0.614 (3.85)	−0.575 (4.05)	−0.610 (4.28)	0.923	0.914	0.981
General health	−0.069 (0.721)	−0.575 (4.05)	−0.095 (0.932)	0.805	0.721	0.799
**HEALTH BEHAVIORS**						
% Exercised (past week)	0.094 (0.465)	0.075 (0.450)	0.122 (0.557)	0.762	0.644	0.620
Communication with MD	0.122 (1.14)	0.201 (1.15)	−0.283 (0.986)	0.922	0.466	*0.023*
Medication adherence	−0.036 (1.01)	−0.074 (1.04)	0.214 (1.18)	0.958	0.682	0.107
**HEALTH-CARE UTILIZATION**						
No. of physician visits	0.150 (3.55)	0.148 (3.82)	0.366 (3.00)	0.925	0.995	0.713
No. ED visits	0.029 (0.602)	−0.113 (0.196)	0.0 (0.392)	*0.046*	*0.032*	0.754
No. of hospital nights	−0.154 (5.77)	0.188 (2.38)	−0.50 (2.39)	0.835	0.696	0.518

*Standard deviations are given in parentheses. *P*-values are from independent sample *t*-tests, except number of ED visits and number of hospital nights, which are from Wilcoxon rank sum tests. *P* (A) compares those who consented (*N* = 246) with all who did not consent (*N* = 281). *P* (B) compares those who consented (*N* = 246) with those who actively declined to consent (*N* = 241). *P* (C) compares those who responded (*N* = 487) with those who did not respond (*N* = 40)*.

*P-values less than 0.05 are shown in italics*.

### Differences between timing of the consent requests

There were 356 participants who were asked to consent after completing the 6-month study period but before being asked to complete 12-month questionnaires. These consisted of all those who had entered the *National Study* in 2011. There were 251 participants who were asked to consent after completing the 12-month follow-up period (those who entered the study during 2010). Of those who were asked to consent after 12 months in the study, 84% had completed 12-month questionnaires. In contrast, only 76% of those who were asked to consent before 12 months eventually completed a 12-month questionnaire (*p* = 0.020 from chi-square).

There were no statistically significant associations between the proportions of participants who consented and when participants were asked. Among those who responded, the proportion who consented was 49.5% for those asked before 12 months and 48.6% for those who had completed the 12-month follow-up period (not shown in tables).

## Discussion

### Results

These data present a unique opportunity to examine factors associated with older adults’ willingness to consent to have their programmatic data linked to administrative claims data. This information is important for identifying potential systematic biases in assessing programmatic impacts using administrative data and guiding future initiatives desiring to link data sources *post hoc*.

The most notable differences between consenters and non-consenters were among the workshop participation variables. Consenters (versus non-consenters) and responders (versus non-responders) attended more sessions and were more likely to complete the program and both 6- and 12-month follow-up questionnaires. This is not unexpected and suggests that those more engaged with the program or with their health-care are more likely to be willing to share their Medicare information.

There were little differences in demographic conditions between the three groups, with the exception of ethnicity. Hispanic and African-Americans were less likely to respond. In contrast, among those who responded, members of these two minority groups were more likely to consent. While non-Hispanic white participants were more likely to respond, they were also more likely to decline to consent.

Although few statistically significant differences in health indicators and health behaviors were found between consenters and non-consenters, participants who consented reported more illnesses or more contact with the medical system. The consenters had higher mean number of self-reported conditions and physician visits at baseline as well as less decreases in ED visits at 6 months than those who did not consent.

While non-responders had a mean of 0.5 days decrease in hospital nights at 6-months, over 50% of the 6-month data was missing for that group. Thus, in our case, any attempt to estimate possible changes in medical expenditures for non-respondents would be subject to bias resulting from the high attrition rate. The likelihood that consenters were both more engaged with their health-care self-management and were likely to have greater numbers of chronic conditions would introduce further bias into studies of Medicare utilization.

### Implications: The consenting process

There is increased concern about the third arm of the Triple Aims for Health-Care, e.g., wanting to document that effective interventions can be provided for better value (14) and lower costs. Thus, cost effectiveness of interventions is becoming more important. To determine costs and cost effectiveness, at least for older adults, examining Medicare claims data is treated as a “gold standard.” To gain such access, participants must usually sign an informed consent. Little is known about the population that consents to examination of their claims data as opposed to those who decline. This study opens a window into these differences.

The best variable for matching data is SSN or at least the last four digits of this number. In our study, only 23% of the initial potential participant population was willing to disclose this number within 3 weeks involving multiple contacts. Even after exhaustive follow-up involving as many as eight attempted contacts, only 42% of the population was willing to consent to having any data used for matching to Medicare data, while 44% actively refused consent.

Of equal importance, we found several significant differences between those who consented and those who did not. Of particular interest are both the baseline differences and 6-month differences in changes in self-reported health-care utilization. If these differences are mirrored in Medicare claims data, it brings to question conclusions regarding the cost effectiveness of these evidence-based interventions. We must acknowledge that such conclusions represent only those who consent and that consenters may represent less than half the population. Furthermore, this population differs in several ways from those who do not consent.

This study highlights limitations in using Medicare or claims data as the sole standard for assessing cost outcomes, if consent is required. Unfortunately, in a free society without a nationalized health service database, it is almost impossible to secure unbiased estimates of costs. It is beyond the scope of this paper to discuss the problems with self-report, billing, or insurance payments. All have well-known problems. We would suggest that the solution to this conundrum is to use two or more methods of estimating costs and triangulating outcomes.

There are at least two other disadvantages for a retrospective consenting process for seniors enrolled in evidence-based programs. First, the personnel costs must be considered. In the case of this study, it took one-and-one-half full-time positions over more than 3 months to attempt to consent just over 600 people.

The second disadvantage is the potential of people opting not to participate in studies, programs, or treatment when consent to examine claims data is required. In the fall of 2013, the Agency for Community Living (ACL) began asking participants in evidence-based community programs funded by the agency to voluntarily consent to having their ZIP Codes and birth dates matched with Medicare data. While it is not known if people did not attend programs because of this request, ACL did receive many complaints from sites and the consenting process was dropped when CMS decided the data would not be needed.

In the study presented here, we estimate that at least 7% of those who had completed 6-month questionnaires and were contacted by phone with a request to consent, both refused to consent and asked to be dropped from the original *National Study* before completing 12-month questionnaires (12 participants). In addition, 6% of the 87 non-respondents refused further contact (5 participants who were put on the do-not-call list) and subsequently did not complete 12-month questionnaires after having completed 6-month questionnaires. Thus, we are aware of at least 17 specific participants in the ongoing study who were likely lost to follow-up as a result of being contacted with a request to consent. Of those who were asked to consent after completion of the 12-month follow-up period, 84% had completed 12-month questionnaires. In contrast, only 76% of those who were asked to consent before 12 months eventually completed a 12-month questionnaire. This suggests that as many as 28 out of 356 participants did not complete 12-month questionnaires and likely would have if they had not been asked to consent. It appears clear that the consenting process contributed to attrition in the larger study. For the *National Study*, where consenting at recruitment was no longer an option, there likely would have had less effect on participation in follow-up questionnaires if we had delayed the consent process for all participants until after all follow-up was completed.

### Limitations

The study to match Medicare data with *National Study* data was conceived and initiated after the *National Study* was well underway. Thus, we lost the opportunity to learn if consent rates might have been different had participants been asked to consent at the time of enrollment in the larger intervention and study. There were little differences in rates of consenting between those who were asked 6 months after entering the study versus those who were asked to consent after the 12-month follow-up period, but it is possible consent rates would have been higher at baseline. However, based on the greater attrition rate among those who were asked to consent before the final follow-up questionnaire, it is likely that asking for consent to match to Medicare data earlier might have reduced participation rates during the initial enrollment in the overall study.

This study was limited to the consenting process and comparing consenters versus others. As noted in the Section “Materials and Methods,” we do not address the actual matching of participant data with CMS Medicare data for those who consented to allow such matching. The matching process is described elsewhere (6). Nor do we attempt to offer solutions for several issues raised. The findings suggest the need for future research on the problem of increased attrition among those asked to allow matching, and on the problem of differences between consenters and non-consenters resulting in bias.

We present a large number of comparisons in Table 1. Because of the exploratory nature of this study, we have not attempted to adjust for multiple comparisons. Thus caution should be exercised in drawing conclusions from any single statistically significant result. Of more importance are the patterns in the results, specifically the tendency of consenters to be more involved with the medical system and to be more involved or engaged in the intervention and larger *National Study*. Further study of the ethnic differences in consenting would be highly desirable.

## Conclusion

Fewer than half the eligible participants consented to link their name, gender, age, and ZIP Code to Medicare data. Those who consented were significantly different in several ways from those who chose not to consent or who did not respond. In particular, consenters may have had more contact with the medical system and more illness. This suggests that data based only on those who consent may be biased toward greater medical utilization and costs. The findings also suggest that asking participants to consent to match Medicare data may reduce participation in an intervention study. These findings have a potential to affect the use of data for policy decisions based on linking Medicare data with specific interventions.

## Conflict of Interest Statement

The authors declare that the research was conducted in the absence of any commercial or financial relationships that could be construed as a potential conflict of interest.

This paper is included in the Research Topic, “Evidence-Based Programming for Older Adults.” This Research Topic received partial funding from multiple government and private organizations/agencies; however, the views, findings, and conclusions in these articles are those of the authors and do not necessarily represent the official position of these organizations/agencies. All papers published in the Research Topic received peer review from members of the Frontiers in Public Health (Public Health Education and Promotion section) panel of Review Editors. Because this Research Topic represents work closely associated with a nationwide evidence-based movement in the US, many of the authors and/or Review Editors may have worked together previously in some fashion. Review Editors were purposively selected based on their expertise with evaluation and/or evidence-based programming for older adults. Review Editors were independent of named authors on any given article published in this volume.
